# Sequential immune-related nephritis and pneumonitis during immune checkpoint inhibitor therapy: a case report

**DOI:** 10.3389/fonc.2026.1804392

**Published:** 2026-05-13

**Authors:** Haocheng Zhao, Shanshan Lin, Lingzhi Wu, Wenqiu Wu

**Affiliations:** 1 Department of Medical Oncology, Yueqing People’s Hospital, Yueqing, Zhejiang, China; 2 Department of Internal Medicine, Yueqing People’s Hospital, Yueqing, Zhejiang, China; 3 Department of Radiology, Yueqing People’s Hospital, Yueqing, Zhejiang, China; 4 Department of Pathology, Yueqing People’s Hospital, Yueqing, Zhejiang, China

**Keywords:** corticosteroid tapering, immune checkpoint inhibitors, immune-related adverse events, immune-related nephritis, immune-related pneumonitis

## Abstract

Immune checkpoint inhibitors (ICIs) have significantly improved cancer treatment but can cause immune-related adverse events (irAEs) that affect multiple organs and may develop over time. We present a case of sequential immune-related nephritis and pneumonitis during ICI therapy. The patient experienced severe acute kidney injury with a considerable increase in serum creatinine level, indicating immune-related nephritis. High-dose corticosteroids led to rapid recovery of kidney function, followed by a gradual taper. However, as the steroid dose was reduced, the patient developed fever and respiratory symptoms, with chest computed tomography revealing signs of immune-related pneumonitis. Prompt re-escalation of corticosteroids resulted in rapid improvement, allowing the taper to be continued slowly. This case highlights the dynamic, sequential nature of irAEs and emphasizes that new immune-mediated toxicities can emerge during steroid tapering even after initial irAE resolution. Therefore, close monitoring and timely therapy adjustments are essential for managing complex irAEs effectively.

## 1 Introduction

Immune checkpoint inhibitors (ICIs), including agents targeting programmed cell death protein 1, programmed death ligand 1, and cytotoxic T-lymphocyte–associated antigen 4, have radically transformed the treatment of many malignancies by restoring effective antitumor immune responses (1). These therapeutic benefits, however, come with the drawback of disrupted immune tolerance, which can cause immune-related adverse events (irAEs) affecting nearly any organ system (1). While many irAEs are mild and easy to manage, some can be severe or even life-threatening (1). Immune-related nephritis, mainly presenting as acute tubulointerstitial nephritis, is an uncommon but clinically important complication, occurring in approximately 2%–5% of patients receiving ICIs (2–4). Similarly, immune-related pneumonitis is rare but one of the most severe irAEs, occurring in approximately 3%–5% of patients and carrying a significant risk of serious morbidity and mortality, especially in severe cases (5, 6). Therefore, current consensus guidelines highlight the importance of early detection and the rapid initiation of systemic corticosteroids for managing moderate to severe irAEs (7). Despite these recommendations, the clinical course of irAEs is still highly variable, and key uncertainties remain about how they develop over time, the optimum duration of immunosuppressive treatment, and the risk of subsequent immune-related toxicities.

Most published reports describe irAEs as isolated incidents or as multiorgan toxicities occurring simultaneously during active ICI therapy exposure (2, 5). In contrast, reports of sequential irAEs impacting different organ systems over time are much less common and not well understood. New clinical observations indicate that immune dysregulation caused by ICIs can continue even after an initial irAE appears to resolve, making patients vulnerable to additional immune-related complications, especially during corticosteroid treatment tapering (7, 8). Immune-related nephritis and pneumonitis share similar immunopathogenic mechanisms and are predominantly caused by T-cell–mediated inflammation and loss of peripheral immune tolerance. Nonetheless, their timing and clinical interaction are not well understood. In this case, we report a patient who initially developed immune-related nephritis and later immune-related pneumonitis during corticosteroid tapering after initial kidney recovery. This case underscores the complex, ongoing nature of irAEs and highlights the importance of continued vigilance and personalized immunosuppressive approaches, even when clinical signs improve.

## 2 Case presentation

A 70-year-old man with gastroesophageal junction adenocarcinoma (Siewert type II) was admitted for evaluation of rapidly worsening renal function during nivolumab-based therapy. He had initially received neoadjuvant combination therapy consisting of nivolumab (360 mg on day 1), oxaliplatin (220 mg on day 1), and capecitabine (1.5 g twice daily on days 1-14) every 3 weeks, followed by radical surgery. Postoperatively, he received adjuvant therapy with nivolumab (360 mg on day 1), oxaliplatin (200 mg on day 1), and S-1 (3 capsules twice daily on days 1-14) every 3 weeks. The patient had no significant past medical history, including no history of chronic kidney disease, autoimmune disease, or other relevant comorbidities, and no long-term use of nephrotoxic medications. The patient had no history of NSAID use before the onset of acute kidney injury. Exposure to proton pump inhibitors was limited to a single administration on the day of chemotherapy at an outside hospital, without prolonged or repeated use before the renal event. After approximately 6 months of nivolumab-based combination therapy, during which he completed a total of six treatment cycles (four neoadjuvant cycles and two postoperative adjuvant cycles) without omission, the patient developed nonspecific fatigue without fever, rash, or urinary symptoms. Routine laboratory tests indicated a significant and sustained increase in serum creatinine level, reaching a peak of 606 μmol/L(normal range: 64–104 μmol/L). Physical examination revealed stable vital signs and no evidence of peripheral edema, skin rash, or joint issues. Urinalysis and renal imaging did not show urinary tract obstruction or infection. Given the close temporal association with ICI exposure and the lack of other explanations, the diagnosis was Common Terminology Criteria for Adverse Events (CTCAE) grade 3 immune-related nephritis causing severe acute kidney injury. A summary of the clinical timeline, laboratory results, and treatment is outlined in **Table 1**.

**Table 1:** A chronological summary of the clinical course, laboratory findings, and management of the case patient over a timeline.

Date	Clinical event	Key findings	Management
2025-10-02	Routine follow-up	SCr 149 µmol/L	Clinical observation
2025-10-07	Progressive renal dysfunction	SCr 183 µmol/L	Continued monitoring
2025-10-10	Transient renal improvement	SCr 106 µmol/L	No intervention
2025-10-18	Severe AKI onset	Peak SCr 606 µmol/L	Diagnosis of irNephritis; initiated IV mPSL (40 mg q8h)
2025-10-20–10-26	Renal recovery phase	Rapid decline in SCr	Continued high-dose corticosteroids
2025-10-29–11-09	Renal function stabilization	Normalization of SCr	Gradual corticosteroid tapering
2025-12-15	New-onset systemic symptoms	Fever and cough	Clinical evaluation
2025-12-18	Pulmonary involvement	Chest CT: Bilateral pulmonary infiltrates	Diagnosis of irPneumonitis; IV mPSL re-escalated (40 mg bid)
2025-12-31	Treatment response	Chest CT: Marked absorption of infiltrates	Continued corticosteroid therapy
2026-01-01–01-15	Recovery and tapering phase	Stable SCr; resolution of respiratory symptoms	Gradual steroid dose reduction
2026–01 onward	Follow-up	No recurrence of nephritis or pneumonitis	Maintenance oral corticosteroids with slow taper

AKI, acute kidney injury; bid, twice daily; CT, computed tomography; irNephritis, immune-related nephritis; irPneumonitis, immune-related pneumonitis; IV, intravenous; mPSL, methylprednisolone; q8h, every 8 hours; SCr, serum creatinine. Normal reference range for serum creatinine: 64–104 μmol/L.

**Figure 1:**
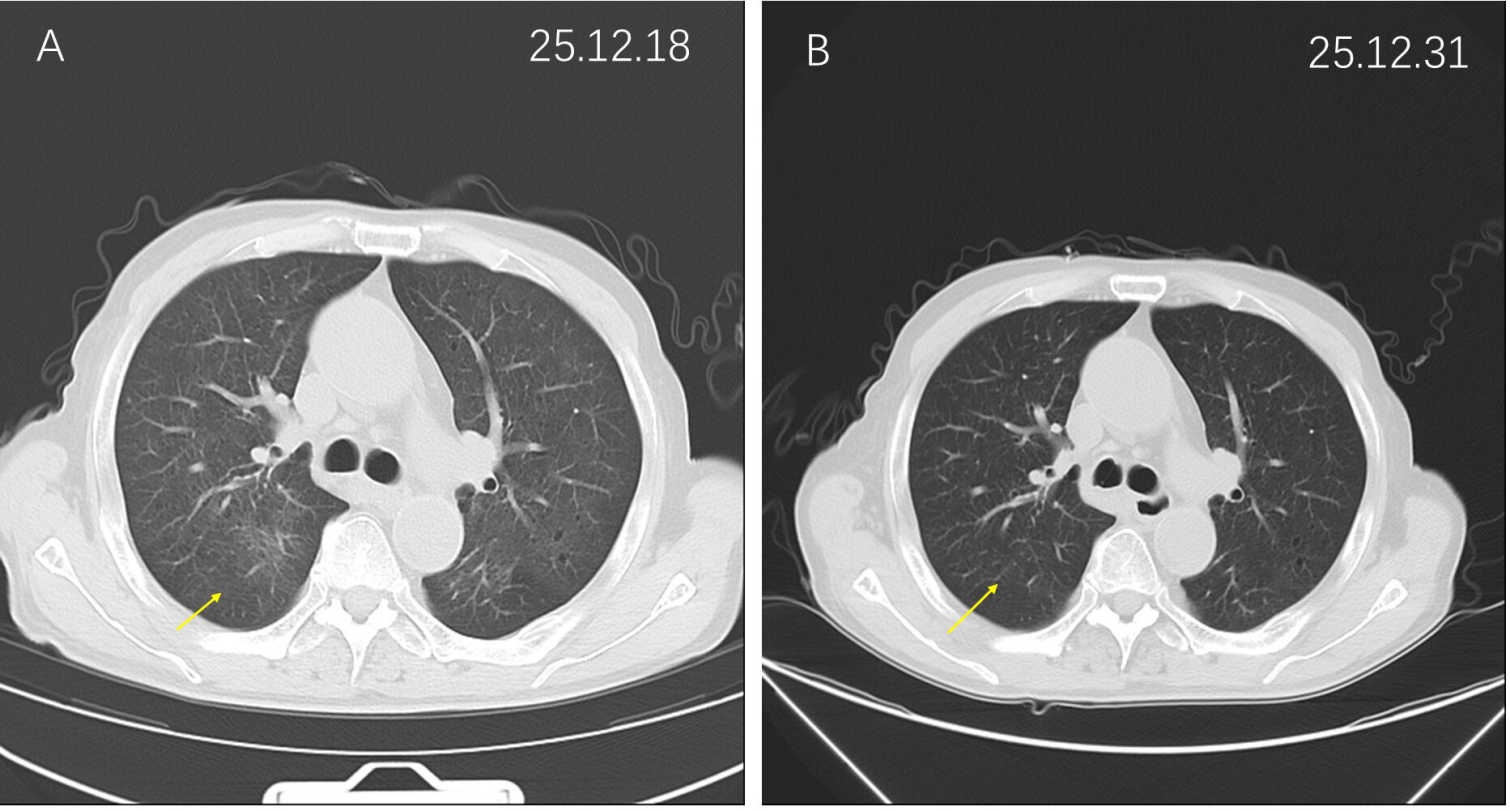
Chest computed tomography (CT) findings of immune-related pneumonitis before and after corticosteroid therapy. Chest CT demonstrated radiographic changes consistent with immune-related pneumonitis and the subsequent treatment response. **(A)** Chest CT at symptom onset during corticosteroid tapering indicated bilateral pulmonary infiltration with patchy ground-glass opacities and interstitial changes, predominantly in the lower lung fields, which was consistent with immune-related pneumonitis. **(B)** Follow-up chest CT performed after corticosteroid re-escalation demonstrated a marked resolution of the previously recorded pulmonary infiltration, with only minimal residual fibrotic changes, indicating a favorable radiographic response to immunosuppressive therapy.

**Figure 2:**
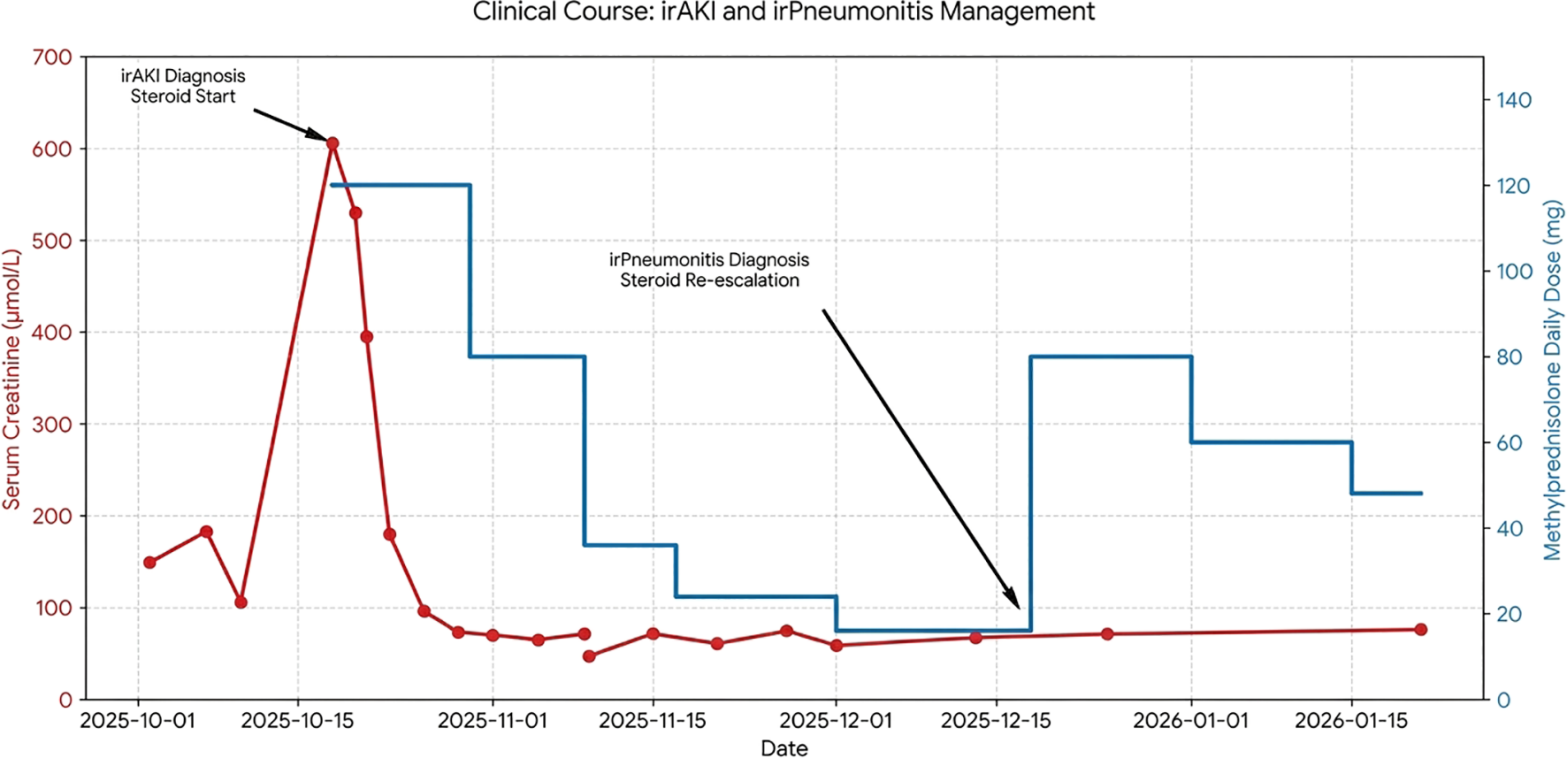
Clinical course of immune-related acute kidney injury and pneumonitis during corticosteroid treatment. Temporal changes in serum creatinine levels (left Y-axis, red line) and daily methylprednisolone dose (right Y-axis, blue line) are shown throughout the entire clinical course. Following immune checkpoint inhibitor therapy, the patient developed severe immune-related acute kidney injury, marked by a rapid increase in serum creatinine, for which high-dose corticosteroid therapy was initiated. Renal function improved promptly and remained stable during subsequent tapering, including during the pneumonitis episode and after corticosteroid re-escalation. During dose reduction, the patient developed immune-related pneumonitis, which necessitated immediate re-escalation of corticosteroid treatment. After steroid intensification, respiratory symptoms and radiographic abnormalities resolved, allowing gradual tapering to continue. Major clinical milestones included the diagnosis of immune-related acute kidney injury, initiation of corticosteroid therapy, the onset of pneumonitis, and steroid re-escalation.

Because of severe immune-related nephritis with a rapid rise in serum creatinine, intravenous methylprednisolone was initiated at 40 mg every 8 hours from October 18 to October 30, 2025. After marked improvement and stabilization of renal function, the dose was tapered to 40 mg twice daily from October 30 to November 9, followed by oral methylprednisolone at 12 mg three times daily from November 9 to November 17, 12 mg twice daily from November 17 to December 1, and 16 mg once daily from December 1 to December 18. The tapering regimen was guided by serial monitoring of renal function and overall clinical stability. When immune-related pneumonitis developed during tapering, intravenous methylprednisolone was promptly re-escalated to 40 mg twice daily on December 18, 2025. After resolution of fever, improvement in respiratory symptoms, and radiographic absorption of pulmonary infiltrates, the corticosteroid dose was gradually tapered again. Approximately 8 months after starting ICI treatment and during the second month of corticosteroid tapering for immune-related nephritis, the patient developed a new fever and cough. Physical examination suggested mild respiratory discomfort without hypoxemia. Chest computed tomography (CT) revealed bilateral pulmonary infiltrates typical of immune-related pneumonitis. During the pulmonary episode, bronchoscopy with bronchoalveolar lavage was recommended to further exclude infectious etiologies, but the patient declined the procedure. Nevertheless, microbiological evaluation was performed, including SARS-CoV-2 PCR, a respiratory viral PCR panel, nasopharyngeal respiratory nucleic acid testing, blood culture, and blood pathogen-targeted testing. No bacterial or fungal pathogen was identified, and no common seasonal respiratory viral pathogen was detected. In particular, both aerobic and anaerobic blood cultures showed no bacterial growth after 5 days of incubation. Although low-level EBV and CMV signals were detected in blood pathogen-targeted testing, these findings were not considered sufficient to explain the acute pulmonary presentation. Therefore, the diagnosis of CTCAE grade 2 immune-related pneumonitis was made based on the overall clinical context, imaging findings, negative infectious workup, temporal association with corticosteroid tapering after ICI therapy, and the subsequent rapid improvement after corticosteroid re-escalation. The microbiological and viral investigations performed during the pulmonary episode are summarized in **Table 2**. Chest CT images before and after corticosteroid re-escalation are depicted in **Figure 1** Corticosteroid therapy was immediately intensified, leading to the rapid resolution of fever and respiratory symptoms. Follow-up chest CT scans showed significant absorption of pulmonary infiltrates, indicating a positive response to treatment. After clinical and radiographic improvements, corticosteroids were gradually tapered and switched to oral form. The timeline of corticosteroid doses, renal function recovery, and the onset and resolution of pneumonitis is illustrated in **Figure 2**. Renal function remained stable thereafter, and during follow-up, no recurrence of pneumonitis or other immune-related side effects was observed. At the latest checkup, the patient was in good health, with sustained renal function and no respiratory issues.

**Table 2:** Microbiological and viral investigations performed during the pulmonary episode.

Date	Test	Specimen	Result	Interpretation
2025-12-14	SARS-CoV-2 PCR	Nasopharyngeal swab	Negative	No evidence of COVID-19 infection
2025-12-15	Respiratory viral PCR panel (13 pathogens)	Nasopharyngeal swab	Negative	No common respiratory viral pathogen detected
2025-12-14	Aerobic blood culture	Blood	No bacterial growth after 5 days	No evidence of bloodstream bacterial infection
2025-12-14	Anaerobic blood culture	Blood	No bacterial growth after 5 days	No evidence of bloodstream bacterial infection
2025-12-19	Respiratory nucleic acid panel (8 pathogens)	Nasopharyngeal swab	Negative	No additional common respiratory viral pathogen detected
2025-12-23	Blood pathogen-targeted testing (200+ panel)	Blood	No bacteria, fungi, parasites, Mycobacterium tuberculosis complex, or non-tuberculous mycobacteria detected; low-level EBV and CMV signals detected	No microbiological evidence supporting bacterial or fungal pulmonary infection; low-level EBV/CMV findings were not considered sufficient to explain the acute pulmonary presentation

## 3 Discussion

irAEs remain among the most challenging aspects of ICI therapy, underscoring the fine balance between achieving strong antitumor immunity and maintaining immune tolerance (1). Although immune-related nephritis and pneumonitis are both well-known toxicities, they are most often reported either as isolated events or as concurrent multiorgan issues during active ICI treatment (2, 5). In contrast, this case is characterized by the sequential development of severe immune-related nephritis followed by immune-related pneumonitis during corticosteroid tapering, after renal function appeared to recover. This timing underscores a serious but often overlooked situation in which immune dysregulation can persist or recur even after the initial irAE seems to be under control.

Immune-related nephritis, most often presenting as acute tubulointerstitial nephritis, is generally responsive to corticosteroid therapy when promptly identified (2, 7). Immune-related pneumonitis, in comparison, is a potentially life-threatening complication that requires early immunosuppressive therapy to avoid serious clinical consequences (5, 6). Although current guidelines emphasize the importance of starting corticosteroids promptly and tapering the dose gradually, practical guidance on the pace and duration of tapering—especially after severe irAEs—remains unclear (7, 9). Reported frequencies of immune-related nephritis and pneumonitis vary across tumor types and immune checkpoint inhibitor classes. In general, immune-related nephritis is uncommon, whereas immune-related pneumonitis is more frequently reported with PD-1/PD-L1 blockade, particularly in combination regimens. To better contextualize the present case, a comparative summary is provided in **Supplementary Table 1**. This case highlights a significant gap in clinical practice by showing that new immune-mediated toxicities can develop during steroid dose reduction, a phase usually viewed as a period of recovery. The sequential effects on the kidney and lung highlight the evolving nature of ICI-induced immune activation and indicate that corticosteroid tapering is a vulnerable period for additional irAEs. Recognizing this pattern is vital for prompt diagnosis and treatment, helping to prevent delays in identifying further immune-related toxicities.

**Figure 3:**
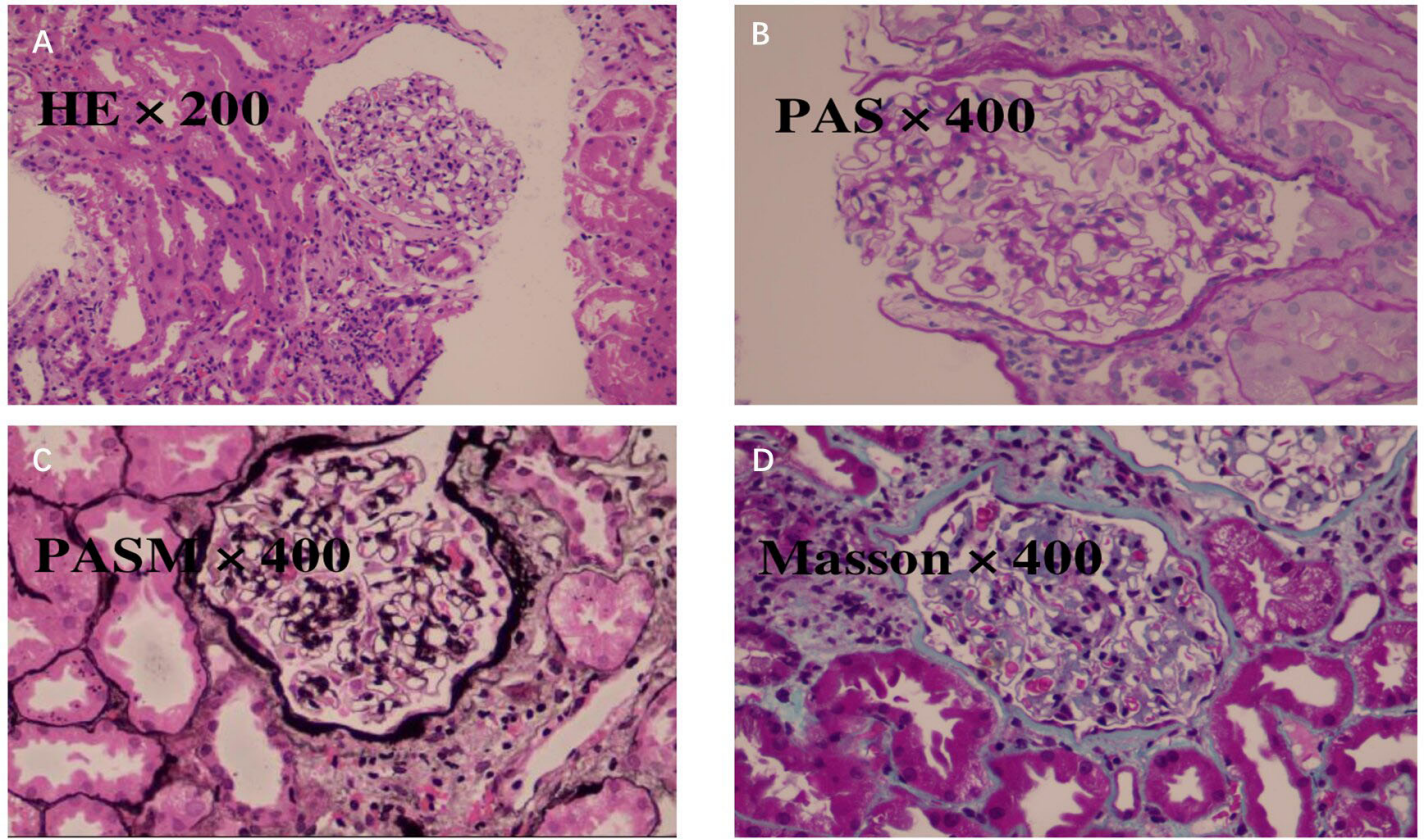
Renal biopsy findings were consistent with immune-related tubulointerstitial nephritis. Renal biopsy revealed histopathological features consistent with immune-related tubulointerstitial nephritis. **(A)** Hematoxylin and eosin (H&E, ×200) staining detected largely preserved glomerular architecture, with focal global glomerulosclerosis affecting a small number of glomeruli. Prominent tubulointerstitial injury was recorded, as characterized by interstitial edema and inflammatory cell infiltration. **(B)** Periodic acid–Schiff (PAS, ×400) staining revealed tubular epithelial cell injury, including vacuolar and granular degeneration, tubular dilation, and a focal loss of the brush border, whereas the glomerular capillary loops remained largely intact. **(C)** Periodic acid–silver methenamine (PASM, ×400) staining demonstrated preserved glomerular basement membrane contours, with no evidence of immune complex deposition or crescent formation. **(D)** Masson trichrome staining (×400) revealed moderate interstitial fibrosis with inflammatory cell infiltration, predominantly lymphocytes and mononuclear cells, which was consistent with active tubulointerstitial inflammation. Immunofluorescence microscopy showed no significant deposition of immunoglobulins or complement components (such as IgG, IgA, IgM, C3, and C1q), and electron microscopy confirmed the absence of electron-dense deposits. Collectively, these findings supported the diagnosis of immune-related acute tubulointerstitial injury.

Accumulating evidence suggests that immune-related nephritis and pneumonitis share a common background of immune dysregulation after immune checkpoint inhibition, including persistent activation of effector CD4+ and CD8+ T cells, impaired peripheral immune tolerance, and reduced regulatory immune control. In addition, peripheral immune cells such as monocytes/macrophages and downstream inflammatory signaling may further amplify tissue injury. However, their organ-specific manifestations differ. Immune-related nephritis most commonly presents as acute tubulointerstitial nephritis characterized by interstitial inflammation and tubular injury, whereas immune-related pneumonitis shows more heterogeneous inflammatory patterns and variable clinical and radiologic features in the lung. These similarities and differences may help explain why distinct organ toxicities can occur sequentially during the course of immune-related adverse events (4–6, 10, 11). Immune-related nephritis typically manifests as acute tubulointerstitial nephritis, marked by interstitial inflammation and tubular damage, whereas glomerular structures remain relatively intact (10, 12). In this case, renal biopsy results (**Figure 3**) showed significant tubulointerstitial inflammation and damage to tubular epithelial cells. No immune complex deposition was evident on immunofluorescence, nor electron-dense deposits on electron microscopy. These findings support a diagnosis of immune-related tubulointerstitial nephritis rather than immune complex–mediated glomerulonephritis. Although ANA and ANCA were not performed, the absence of a prior autoimmune history together with the biopsy findings, negative immunofluorescence, and absence of immune complex deposition or electron-dense deposits supported a tubulointerstitial rather than immune complex-mediated process. Such pathological features align with previous reports that describe ICI-associated renal injury as mainly T cell–mediated (2, 10, 13). Renal biopsy represented a major diagnostic strength in this case. Histopathological examination showed acute tubulointerstitial injury with interstitial inflammation, whereas immunofluorescence was negative and no immune complex deposition or electron-dense deposits were identified. These findings are consistent with the biopsy pattern most commonly reported in ICI-related nephrotoxicity, particularly acute tubulointerstitial nephritis, and support a T cell-mediated process rather than an immune complex-mediated glomerular disease. In addition, the diagnostic reasoning in this case was based on a process of elimination rather than temporal association alone. For the renal event, common drug-related confounders were considered less likely because there was no NSAID exposure and no prolonged PPI use before the onset of acute kidney injury. Capecitabine-induced nephrotoxicity was considered less likely because capecitabine was not part of the most recent treatment immediately preceding the onset of acute kidney injury, and the renal biopsy findings were more consistent with immune-related tubulointerstitial nephritis. Oxaliplatin-induced lung injury was also considered in the differential diagnosis of the subsequent pulmonary event. However, it was considered less likely because the pulmonary abnormalities developed during corticosteroid tapering after biopsy-supported immune-related nephritis, extensive microbiological and viral investigations did not support infection, and the findings improved rapidly after corticosteroid re-escalation. Nevertheless, oxaliplatin-induced lung injury could not be excluded with absolute certainty. Autoimmune causes could not be excluded serologically because ANA and ANCA were not performed; however, the absence of a prior autoimmune history together with the biopsy pattern made a primary immune complex-mediated autoimmune process less likely. For the subsequent pulmonary event, bronchoalveolar lavage was not performed because the patient declined the procedure, which remains an important limitation. Nevertheless, repeated microbiological and viral investigations did not identify a bacterial, fungal, or common seasonal respiratory viral pathogen, and the pulmonary findings improved rapidly after corticosteroid re-escalation. Taken together, these findings support the interpretation that the nephritis and subsequent pneumonitis were most consistent with sequential ICI-related toxicities. Immune-related pneumonitis, despite its variable clinical and radiologic features, involves immune-driven inflammation of target organs and often requires prompt immunosuppressive therapy to prevent complications (5, 6, 11, 14). Although both entities are well recognized individually, their temporal relationship remains poorly understood. Growing clinical evidence suggests that irAEs may evolve sequentially rather than in isolation, reflecting persistent immune dysregulation even after the apparent resolution of an initial event (8). Corticosteroid tapering has emerged as a particularly vulnerable period during which incomplete immune suppression or immune rebound may precipitate additional irAEs (7, 8). However, reliable predictors for these sequential events are not well established, which limits clinicians’ guidance during recovery. This case contributes to the literature by showing that immune-related pneumonitis can occur during corticosteroid tapering following biopsy-confirmed immune nephritis. It supports the idea that irAEs are a dynamic and evolving spectrum, rather than isolated, self-limited issues.

In summary, this case enhances our understanding of irAEs by highlighting that they are a dynamic and evolving process rather than isolated, self-limiting events. The literature consistently recommends initiating corticosteroids promptly for moderate to severe irAEs and then gradually tapering them once symptoms improve (7). Our findings align with these principles, as both immune-related nephritis and pneumonitis exhibited rapid improvement with increased steroid doses. However, this case questions the routine clinical belief that corticosteroid tapering always indicates recovery. Instead, it suggests that tapering might reveal hidden immune dysregulation and trigger new organ-specific irAEs, even after the initial toxicity appears to be resolved. Although delayed or sequential irAEs are occasionally reported, they remain underrecognized in everyday clinical practice (8). From a clinical standpoint, this case underscores the importance of careful corticosteroid tapering, a crucial phase that requires ongoing vigilance, especially in patients with a history of severe irAEs. Regular monitoring for new systemic or organ-specific symptoms, prompt laboratory and imaging assessments, and maintaining a low threshold for increasing immunosuppression can aid in early detection and minimize morbidity (4–7, 9, 11). To further summarize the practical implications of this case, a proposed follow-up and management framework during corticosteroid tapering is provided in **Table 3**. The patient’s positive outcome highlights that adaptable and prompt corticosteroid adjustments can effectively manage sequential irAEs without causing permanent organ damage. By treating corticosteroid tapering as a sensitive period, this case offers valuable clinical insights that support a personalized and flexible approach to irAE management in patients receiving ICIs.

**Table 3:** Proposed follow-up and management considerations during corticosteroid tapering after severe irAEs.

Clinical domain	Proposed consideration	Clinical rationale
Symptom monitoring	Closely monitor for new systemic or organ-specific symptoms, especially fever, cough, dyspnea, fatigue, or reduced urine output.	New or sequential irAEs may emerge during corticosteroid tapering and can initially present with nonspecific symptoms.
Laboratory and imaging reassessment	Repeat laboratory tests and obtain imaging promptly when new symptoms or suspicious findings arise.	Early reassessment may facilitate timely recognition of recurrent or newly emerging irAEs.
Differential diagnosis	Reassess for infection, tumor progression, and other treatment-related toxicities.	Alternative causes should be excluded before attributing new findings to irAEs.
Corticosteroid management	Maintain a low threshold for slowing the taper or re-escalating corticosteroids when new immune-mediated toxicity is suspected.	Corticosteroid tapering may be a vulnerable period for persistent or rebound immune dysregulation.
Multidisciplinary evaluation	Consider timely multidisciplinary input according to the organs involved.	Coordinated assessment may improve the diagnosis and management of sequential or multiorgan irAEs.

irAE, immune-related adverse event.

## 4 Conclusions

This case underscores the dynamic and evolving nature of irAEs linked to ICI therapy. Even after an initial toxicity appears to resolve, subsequent involvement of other organs might occur, especially during corticosteroid tapering. Instead of signifying full recovery, reducing steroid doses may mark a vulnerable phase where immune dysregulation becomes clinically noticeable. Therefore, careful clinical monitoring, along with timely laboratory tests and imaging, is crucial throughout this stage. Importantly, early detection of new immune-related toxicities and adaptable immunosuppressive treatment can result in positive outcomes without permanent organ damage. Overall, these findings indicate the need for a flexible, personalized approach to managing irAEs, emphasizing ongoing vigilance beyond initial improvement to ensure patient safety and maximize the benefits of ICIs.

## Data Availability

The original contributions presented in the study are included in the article/**Supplementary Material**. Further inquiries can be directed to the corresponding author.
