# Supplementary material for: Sequential immune-related nephritis and pneumonitis during immune checkpoint inhibitor therapy: a case report
Source: Front Oncol. 2026 May 13;16:1804392. doi: 10.3389/fonc.2026.1804392 (PMC13212114; doi:10.3389/fonc.2026.1804392)
Supplement: Supplementary file 1 [file Table1.pdf]

Supplementary Table S1.

Reported frequencies of immune-related nephritis and pneumonitis across selected immune checkpoint inhibitor classes and tumor settings

Tumor type / setting	ICI class / regimen	irNephritis	irPneumonitis	Reference
Mixed solid tumors	Anti–PD-1 monotherapy	1.4%–3%	2.7%–3.0%	Sprangers et al.; Nishino et al.
Mixed solid tumors	Anti–PD-L1 monotherapy	<1%	~1.3%	Seethapathy et al.; Khunger et al.
Mixed solid tumors	Anti–CTLA-4 monotherapy	Uncommon; variably reported in pooled monotherapy analyses	Usually <1%	Sprangers et al.; review articles
Mixed solid tumors	Anti–PD-1 + anti–CTLA-4 combination	Up to 5%	6.6%–10.0%	Sprangers et al.; Nishino et al.; Naidoo et al.
Melanoma	Anti–PD-1/PD-L1–based therapy	NR	Lower than in NSCLC; increased with combination therapy	Naidoo et al.
NSCLC	Anti–PD-1/PD-L1–based therapy	NR	Higher than in melanoma; in one meta-analysis, ~3.6% with PD-1 vs ~1.3% with PD-L1	Naidoo et al.; Khunger et al.
Gastroesophageal junction adenocarcinoma (present case)	Nivolumab-based combination therapy	Case-based	Case-based	Present case

Abbreviations: ICI, immune checkpoint inhibitor; irNephritis, immune-related nephritis; irPneumonitis, immune-related pneumonitis; NR, not specifically reported; NSCLC, non-small cell lung cancer.

Note: Reported frequencies vary across studies because of differences in tumor type, treatment regimen, study design, and adverse-event definitions. This table is intended to provide concise clinical context for the present case rather than an exhaustive systematic review.
